# Phylogenetic relationships of *Zieria* (Rutaceae) inferred from chloroplast, nuclear, and morphological data

**DOI:** 10.3897/phytokeys.44.8393

**Published:** 2015-01-13

**Authors:** Cynthia M. Morton

**Affiliations:** 1 Head of Section of Botany, Carnegie Museum of Natural History, 4400 Forbes Avenue, Pittsburgh, Pennsylvania 15213-4080, U.S.A.

**Keywords:** *Zieria*, Rutaceae, Boronieae, Australia, conservation

## Abstract

*Zieria* Sm. (Rutaceae, Boronieae) is predominantly native to eastern Australia except for one species, which is endemic to New Caledonia. For this study, sequence data of two non-coding chloroplast regions (*trnL-trnF*, and *rpl32-trnL*), one nuclear region (ITS region) and various morphological characters, based on Armstrong’s (2002) taxonomic revision of *Zieria*, from 32 of the 42 described species of *Zieria* were selected to study the phylogenetic relationships within this genus. *Zieria* was supported as a monophyletic group in both independent and combined analyses herein (vs. Armstrong). On the basis of Armstrong’s (2002) non-molecular phylogenetic study, six major taxon groups were defined for *Zieria*. The Maximum-parsimony and the Bayesian analyses of the combined morphological and molecular datasets indicate a lack of support for any of these six major taxon groups. On the basis of the combined Bayesian analysis consisting of molecular and morphological characters, eight major taxon groups are described for *Zieria*: 1. *Zieria cytisoides* group, 2. *Zieria granulata* group, 3. *Zieria laevigata* group, 4. *Zieria smithii* group, 5. *Zieria aspalathoides* group, 6. *Zieria furfuracea* group, 7. *Zieria montana* group, and 8. *Zieria robusta* group. These informal groups, except for of the groups *Zieria robusta* and *Zieria cytisoides*, correspond to the clades with posterior probability values of 100.

## Introduction

*Zieria* Sm. (Rutaceae, Boronieae) comprises 42 species. Six major taxonomic groups were defined based on non-molecular characters, according to the most recent classification by Armstrong (2002). Within Armstrong’s (2002) tribal concept of the Boronieae, *Zieria* forms a distinct clade with *Boronia* Sm. s. l., *Boronella* Baill., *Brombya* F. Muell., *Medicosma* Hook.f., *Neobyrnesia* J.A. Armstr. and *Euodia* J.R. Forst. & G. Forst. s. s.; this clade is characterized by the presence of foliar sclereids.

*Zieria* consists of prostrate shrubs to small trees, with opposite and trifoliolate, or rarely unifoliolate leaves. Inflorescences are axillary, with four-merous, white or pink flowers. The fruits are comprised of one to four basally connate cocci, which dehisce explosively along the adaxial and apical margins. The seeds are usually one (often by abortion of one ovule) per fruit, with a thin brittle testa that is irregularly sculptured. In general, *Zieria* is distinguished from other genera of the Australian Rutaceae by the combination of opposite leaves, the conspicuous and 4-merous flowers, free petals, four stamens, free filaments, a deeply four-lobed disc, and dry, dehiscent fruits. This genus is predominantly native to eastern Australia, with the exception of the one species, *Zieria chevalieri* Virot., which is endemic to New Caledonia. The distribution in eastern Australia extends from northeastern Queensland to Tasmania and as far west as Kangaroo Island in South Australia.

Sir James E. Smith first described the genus in 1798, in memory of Jan Zier, a Polish botanist. In 1810, H.C. Andrews described the first species, *Zieria smithii* Andrews, in H.C. Andrew’s Botanist’s Repository. In 1815, Bonpland published the descriptions of four species and soon after, in 1818, J.E. Smith described five more species. Bentham in his Flora Australiense (1863) described 11 new taxa and provided the first comprehensive key, with descriptions, synonyms and distribution data. For almost 136 years very little taxonomy was completed apart from C.T. White’s descriptions of five new taxa in 1942, and Virot’s (1953) circumscription of the endemic species from New Caledonia. It was not until 2002 that Armstrong reassessed and revised the classification, including defining six major taxonomic groups within *Zieria*. Accordingly, the nomenclature used in this paper is that of Armstrong (2002) and incorporates the morphological phylogenetic characters from that study (cf. Table 1). This study will be the first to test the monophyly of *Zieria* and its six major taxonomic groups using molecular data.

**Table 1.** The six taxonomic groups within *Zieria* as defined by Armstrong (2002).

*Zieria*, Group A
*Zieria adenodonta* (F. Muell.) J.A. Armstr.
*Zieria adenophora* Blakely
*Zieria buxijugum* J.D. Briggs & J.A. Armstr.
*Zieria collina* C.T. White
*Zieria floydii* J.A. Armstr.
*Zieria formosa* J.D. Briggs & J.A. Armstr.
*Zieria furfuracea* R.Br. ex Benth.
*Zieria granulata* C. Moore ex Benth.
*Zieria hindii* J.A. Armstr.
*Zieria obcordata* A. Cunn.
*Zieria parrisiae* J.D. Briggs & J.A. Armstr.
*Zieria robusta* Maiden & Betche
*Zieria tuberculata* J.A. Armstr.
*Zieria verrucosa* J.A. Armstr.
*Zieria*, Group B
*Zieria arborescens* Sims
*Zieria caducibracteata* J.A. Armstr.
*Zieria lasiocaulis* J.A. Armstr.
*Zieria oreocena* J.A. Armstr.
*Zieria southwelli* J.A. Armstr.
*Zieria*, Group C
*Zieria chevalieri* Virot
*Zieria fraseri* Hook.
*Zieria laevigata* Bonpl.
*Zieria laxiflora* Domin
*Zieria*, Group D
*Zieria montana* J.A. Armstr.
*Zieria prostrata* J.A. Armstr.
*Zieria robertsiorum* J.A. Armstr.
*Zieria smithii* Andrews
*Zieria*, Group E
*Zieria aspalathoides* A. Cunn. ex Benth.
*Zieria citriodora* J.A. Armstr.
*Zieria ingramii* J.A. Armstr.
*Zieria minutiflora* (F. Muell.) Domin
*Zieria obovata* (C.T. White) J.A. Armstr.
*Zieria odorifera* J.A. Armstr.
*Zieria pilosa* Rudge
*Zieria rimulosa* C.T. White
*Zieria*, Group F
*Zieria baeuerlenii* J.A. Armstr.
*Zieria covenyi* J.A. Armstr.
*Zieria cytisoides* Sm.
*Zieria involucrata* R.Br. ex Benth.
*Zieria littoralis* J.A. Armstr.
*Zieria murphyi* Blakely
*Zieria veronicea* (F. Muell.) Benth.

A subfamilial phylogenetic analysis was completed for Rutaceae by Chase et al. (1999), Groppo et al. (2008, 2012), Poon et al. (2007), Bayly et al. (2013), and Morton and Telmer (2014), using evidence from *rbcL* and *atpB*, *rps16* and *trnL-trnF* and *trnL-F*, *xdh*, and ITS sequence variation. All of the above authors, except for Bayly et al. (2013), did not include taxa from either *Zieria* or *Neobyrnesia* (sister genus to *Zieria*). Bayly et al. (2013) only included three *Zieria* species and *Neorbyrnesia*, and therefore, their relationships to each other and to other taxa of Rutaceae based on molecular techniques need to be examined for the degree of congruence with morphological characters. Of the 32 species used in this study, 21 are considered endangered or vulnerable according to the Environment Protection and Biodiversity Conservation (EPBC) Act (http://www.environment.gov.au/cgi-bin/sprat/public/spratlookupspecies.pl?name=zieria&searchtype=Wildcard).

Molecular studies can produce effective and practical solutions for conservation biology to taxonomic uncertainties with respect to rare and threatened taxa and, in light of the high proportion of endangered taxa and overlying distribution patterns for a number of these taxa, examinations should be conducted on *Zieria*.

The goals of this study are (1) to test the monophyly of the genus *Zieria* and to identify its closest relatives; (2) to evaluate the six taxonomic groups within *Zieria* as recognized in the most recent revision (Armstrong 2002); and (3) to examine the relationship based on distribution patterns and molecular change of the endangered or vulnerable taxa of *Zieria*.

## Methods

For this study, two non-coding chloroplast regions (*trnL-trnF*, and the *rpl32-trnL*) were selected, as well as the Internal Transcribed Spacer (ITS) of the nuclear region and various morphological characters. The *trnL-trnF* region consists of the *trnL* intron and the *trnL-trnF* intergenic spacer (Taberlet et al. 1991). The *rpL32-trnL* intergenic spacer is in the SSC (small single copy) region of the chloroplast genome. The *rpl32-trnL* was first used for phylogenetic studies by Shaw et al. (2005). Various workers have found that both of these sequences provided good resolution at the generic and species level (e.g. Wallander and Albert 2000; Baker et al. 2000). The ITS region of the *18S-26S* nuclear ribosomal DNA (nrDNA) consists of three genes that code for the *18S*, *5.8S* and *26S* ribosomal subunits. The three genes are separated by two internal transcribed spacers, ITS1 between *18S* and *5.8S* and ITS2 between *5.8S* and *26S*. Morphological characters were taken from information in Armstrong’s (2002) taxonomic revision of *Zieria* (Table 1).

### Taxon sampling & DNA extraction

Vouchers for the 33 species used in this study along with the GenBank accession numbers are listed in the Appendix 1. The total genomic DNA was extracted from (0.5—1.0 g) fresh or dried leaf material. Leaves were ground with a mortar and pestle and subsequently treated with the DNEasy plant DNA extraction kit from Qiagen (Qiagen, Valencia, California, USA) following the manufacturer’s protocol. Alignments were made using the Sequencher software program (Gene Codes Corporation, Ann Arbor, MI), for each marker for 32 *Zieria* and 1 *Neobyrnesia* species and also a broader *trnL-F* alignment with sampling across all Rutaceae subfamilies including Meliaceae and Simaroubaceae as outgroups. All GenBank accessions numbers for the additional sequences can be found in Morton and Telmer (2014) with the exception of *Boronia* (EU853780), and *Medicosma* (EU853806) and *Euodia* (EU493243).

### rpl32-trnL

The *rpl32-trnL* gene in 33 species was amplified using the primer pair *rpl32F/trnL* (Shaw et al. 2005) to acquire the entire region. The final PCR cocktail of 50 μl contained the following: 38 µl water, 5 µl of 10% Mg free buffer solution, 3 µl of 25 mM MgCl_2_, 1 µl of 10 mM dNTPs, 0.25 µl *Taq* polymerase, and 0.5 µl of each primer. The amplifying reactions were run for 25 cycles of denaturing for 30 s at 95 °C, primer annealing for 50 s at 57 °C, and elongation for 2 min at 72 °C.

### trnL-trnF

The *trnL* intron and the *trnL-trnF* intergenic spacer for 33 species were PCR-amplified using the universal primers trn-c, trn-d, trn-e, and trn-f as described by Taberlet et al. (1991). For some samples the entire *trnL* intron/*trnL-trnF* spacer region was amplified with *trn-c* and *trn-f*. In others, two separate amplifications were performed, one to amplify the *trnL* intron with trn-c and trn-d and the other to amplify the *trnL-trnF* spacer with *trn-e* and *trn-f*. In general each 50 µl amplification reaction contained the same proportions as in the *rp16* reactions. PCR amplification used a 7-min denaturing step at 94 °C followed by 30 cycles of denaturing for 1 min at 94 °C, primer annealing for 1 min at 45 °C, and elongation for 1 min at 72 °C, with a final 7-min elongation step at 72 °C.

### ITS

The amplification of the ITS was performed successfully on 33 species using oligonucleotide primers ITS1/ITS4 (White et al. 1990) to acquire the entire region. The DNA fragment amplified using these two primers is approximately 800 bp long and includes ITS1, ITS2 and the *5.8S* ribosomal gene. The basic mix contained the following: 38 µl of water, 5 µl of 10% Mg free buffer solution, 3–6 µl of 25 mM MgCl_2_, 1 µl of 10 mM dNTPs, 0.5 µl of each primer (10 nM), and 0.25 µl *Taq* DNA along with 1.5 µl of DNA temfig for each reaction. The thermal cycler was programmed to perform an initial 1 cycle of denaturation at 95 °C for 2 min, followed by 24 cycles of 30 seconds at 55 °C, 72 °C for 90 seconds and 95 °C for 30 seconds. This was followed by a 10 min. extension at 72 °C to allow completion of unfinished DNA strands.

### Cycle sequencing

The PCR products were cleaned using the QIAGEN QIAquick PCR purification kit (QIAGEN Inc., Chatsworth, California, USA) following the protocols provided by the manufacturer. Cleaned products were then directly sequenced using the ABI PRISM Dye Terminator Cycle Sequencing Ready Kit with AmpliTaq DNA Polymerase (Applied Biosystems Inc., Foster City, California, USA). Unincorporated dye terminators were removed using the QIAGEN DyeEx dye-terminator removal system (QIAGEN Inc.) following the manufacturer’s recommendations. Samples were then loaded into an ABI 3100 DNA Sequencer. The sequencing data was analyzed and edited using the Sequencher software program (Gene Codes Corporation, Ann Arbor, Michigan, USA).

### Morphological characters

A morphological dataset of 48 characters was constructed. Twenty-eight characters were coded as unordered binary and 20 as multistate. All but two characters (4-types of pubescence on young branches and 12-presence or absence of revolute lamina margins) were variable within *Zieria*. The invariant characters were included because they were thought to be important in testing the monophyly of the genus. All analyses were conducted as stated in the analysis section. Character states of taxa were taken from Armstrong (2002: 291–294).

### Phylogenetic analysis

Boundaries of the *trnL* intron, *rpl32-trnL*, and the ITS nuclear gene were determined by comparison with sequences in GenBank. The following two alignment criteria and methodology were used: (1) when two or more gaps were not identical but overlapping, they were scored as two separate events and (2) phylogenetically informative indels (variable in two or more taxa) were scored as one event at the end of the data set. All DNA sequences reported in the analyses have been deposited in GenBank (Appendix 1).

Maximum-parsimony (MP) analyses of all single markers as well as the combined datasets were performed in PAUP* 4.0b8 (Swofford 2002) using the heuristic search option and with uninformative characters excluded. Searches were conducted with 100 random-taxon-addition replicates with TBR branch swapping, steepest descent, and MulTrees selected with all characters and states weighted equally and unordered. All trees from the replicates were then swapped onto completion, all shortest trees were saved, and a strict consensus or majority rule tree was computed. Relative support for individual clades was estimated with the bootstrap method (Felsenstein 1985). One thousand pseudoreplicates were performed with uninformative characters excluded. Ten random-taxon-addition heuristic searches for each pseudoreplicate were performed and all minimum-length trees were saved for each search. To reduce bootstrap search times, branches were collapsed if their minimum length was zero (“amb-“).

The Bayesian analysis of the combined molecular and morphological analysis used a mixed-model approach (Mr Bayes 3.1.2 Ronquist et al. 2005). MrModelTest v2.3 (Posada and Crandall 1998, 2001; Nylander 2004) was used to choose the best evolutionary model, as selected by the Akaike Information Criterion. Four independent analyses were run, each performing 10 million generations, sampling every 1000^th^ generation and using 3 heated and 1 cold chain, and other default settings. Tracer v1.4.1. (Rambaut and Drummond 2007) was used to assess convergence of the runs and to discard the initial 20% of the trees as a burn-in. Branch lengths are averaged from the distribution of trees and the posterior probability values (BPP) for the branches reported (Nylander et al. 2004). Morphological state changes were examined on the combined tree by using MacClade 4.0 (Maddison and Maddison 2000).

To determine the combinability of the data sets, their data structure was compared using methods outlined by Mason-Gamer and Kellogg (1996), who discussed various ways to assess conflict between data sets. In one method the combination of independent data sets is possible if the trees do not conflict or if conflict receives low bootstrap support. Therefore, each node on the independent trees is tested for congruence against the other. If the nodes do not contain conflicting information, they are congruent and the data sets are combinable. Where there are incongruent nodes, the bootstrap values for each node are examined. If the support is less than 70%, there is no hard conflict and the incongruence is interpreted as being due to chance. In this study the different data sets were analyzed in combination to see how each data set changed or confirmed the tree topologies of each other and to adopt a hypothesis of phylogenetic relationships for the genus.

### Conservation

Morton and Schlesinger (2014) found that species with low genetic diversity are less able to respond to environmental change; therefore this information can be informative and has been considered.

This study examined the following 15 of the 21 endangered or vulnerable species (*Zieria adenophora*, *Zieria baeuerlenii*, *Zieria buxijugum*, *Zieria citriodora*, *Zieria collina*, *Zieria convenyi*, *Zieria formosa*, *Zieria granulata*, *Zieria ingramii*, *Zieria murphyi*, *Zieria obcordata*, *Zieria parrisiae*, *Zieria prostrata*, *Zieria verrucosa*, and *Zieria tuberculata*). An examination for similarity was made using the distribution patterns and the number of bp changes within all three genes for the taxa in clades that had strong posterior probabilities.

## Results

The inclusion of gap coding in all data sets containing molecular data resulted in more homoplasy and lack of resolution; therefore, gap coding was not used in the following results. GenBank sequences EU281855–EU281953 were specifically generated for this study.

### Larger trnL-trnF Family Analysis

**Figure 1.**
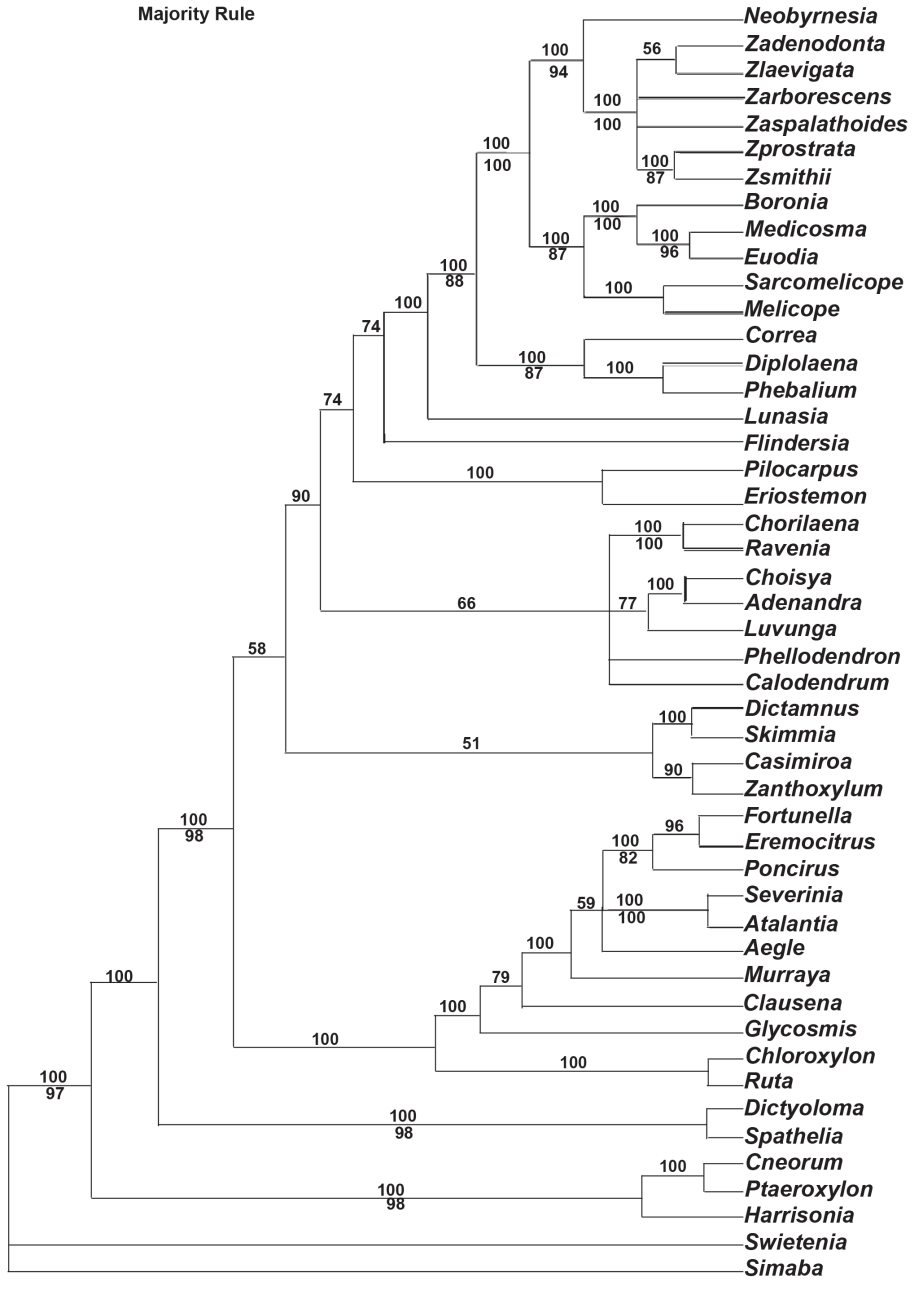
MP majority rule consensus tree of the expanded *trnL-trnF* dataset using a broad sampling of genera of Rutaceae as well as *Simaba* (Simaroubaceae) and *Swietenia* (Meliaceae) as outgroups. Numbers below nodes are bootstrap values.

Multiple sequence alignment of *Zieria* and *Neobyrnesia* with 44 other Rutaceae and closely related taxa resulted in a data matrix of 1038 characters. No regions were excluded. Of the 1038 positions constituting the aligned *trnL-trnF* sequences, 357 (34%) were variable and 408 (39%) were parsimony-informative. The analysis recovered 4,383 equally optimal trees of 1037 steps (CI = 0.57, RI = 0.72; Fig. 1).

*Zieria* are supported as a monophyletic clade in the strict consensus tree (BS 100%). Sister to six species of *Zieria* is the genus *Neobyrnesia* (BS 94%). Sister to this grouping is (((*Medicosma* and *Euodia* (BS 96%)) *Boronia* (BS100%)) (*Sarcomelocope* and *Melicope* (BS 100)) (BS 87%)) followed by the remaining taxa. *Neobyrnesia* was therefore selected as the outgroup for this study.

### trnL-trnF

Multiple sequence alignment of *Zieria* and *Neobyrnesia* resulted in a matrix of 1035 characters. A total of 10 gaps were required for proper alignment of the *trnL-trnF* sequences. These gaps ranged from one to 15 bps. No regions were excluded. Mean percentage G + C content was 56%. Of the 1035 positions, 127 (12.3%) were variable and 33 (3.2%) were parsimony-informative. The analysis recovered 35,458 equally optimal trees of 71 steps (CI = 0.59, RI = 0.69).

*Zieria* was supported as monophyletic in the strict consensus trees (BS 100). Most of *Zieria* consists of an unsupported grade or small polytomies except for one minor clade with bootstrap support of 75% (*Zieria furfuracea* R.Br. ex Benth. and *Zieria laxiflora* Domin).

### Rpl32-trnL

Multiple sequence alignment of *Zieria* and *Neobyrnesia* resulted in a matrix of 1180 characters. Approximately 14 gaps were required for proper alignment of the *rpL32-trnL* sequences. These gaps ranged from one to 49 bps. No regions were excluded. Mean percentage G + C content was 30%. Of the 1180, 236 (20%) were variable and 46 (3.9%) were parsimony-informative. The analysis recovered 87,213 equally optimal trees of 77 steps (CI = 0.69, RI = 0.90).

*Zieria* was supported as monophyletic in the strict consensus trees (BS 100). The tree mainly consists of a polytomy except for one minor clade with bootstrap support greater than 75% (*Zieria furfuracea* and *Zieria laxiflora* (BS 95%)).

### ITS

Multiple sequence alignment of *Zieria* and *Neobyrnesia* resulted in a data matrix of 714 characters. Approximately five gaps were required for proper alignment of the ITS sequences. These gaps ranged from one to 16 bps. No regions were excluded. Mean percentage G + C content was 36%. Of the 714, 207 (29%) were variable and 82 (11.5%) were parsimony-informative. The analysis recovered 7,259 equally optimal trees of 169 steps (CI = 0.72, RI = 0.84). *Zieria* is supported as a monophyletic clade in the strict consensus tree (BS 100%). Basal within this clade is *Zieria citriodora* J.A. Armstr., which is sister to *Zieria aspalathoides* A. Cunn. Ex Benth. and *Zieria ingramii* J.A. Armstr. (BS 88%). The backbone phylogeny of the genus remained unresolved, however a number of minor clades were inferred. Clades that contain bootstrap support greater than 75% starting from the base of the tree include: 1) a clade containing *Zieria arborescens* Sims sister to a polytomy of *Zieria covenyi* J.A. Armstr., *Zieria murphyi* Blakely and *Zieria odorifera* J.A. Armstr. (BS 88%); 2) a clade containing *Zieria montana* J.A. Armstr. and *Zieria southwelli* J.A. Armstr. (BS 100%); 3) a clade containing a polytomy of *Zieria adenophora* Blakely, *Zieria furfuracea* and *Zieria laxiflora* (BS 100%); 4) a clade containing *Zieria fraseri* Hook. and *Zieria laevigata* Bonpl. (BS 100%); 5) a clade containing *Zieria pilosa* Rudge and *Zieria verrucosa* J.A. Armstr. (BS 100%); and 6) a clade containing ((*Zieria collina* C.T. White and *Zieria prostrata* J.A. Armstr. (BS 89%)) sister to *Zieria adenodonta* (F. Muell.) J.A. Armstr. (BS 77%)).

### Phylogenetic utility of the three genes (*trnL-trnF*, *rpl32-trnL*, and ITS) in *Zieria*

The respective numbers of variable and potentially phylogenetically informative characters in each dataset, the consistency indices and the numbers of branches with bootstrap support above 75% can be found in Table 2. The ITS sequences produced the most parsimony-informative characters for similar taxon sampling when compared with the other regions: *trnL-trnF* (33), *rpl32-trnL* (46), and ITS (82). The *trnL-trnF* gene produced the fewest parsimony-informative characters. The ITS gene also had the highest number of resolved nodes at or above 75% bootstrap support when compared with all other genes: *trnL-trnF* (2), *rpl32-trnL* (2), and ITS (9). The combined parsimony analysis had 7 nodes at or above 75% bootstrap support whereas in the Bayesian analyses 13 branches had posterior probability values higher than 93%. There was no correlation between the increase of the CI and RI values and the increase in the number of informative characters.

**Table 2.** Genetic statistics for genes and genic regions utilized in the individual genic analyses, and in the combined molecular and morphological datasets.

Results	trnL	rpl32	ITS	molecular	morphology	Total data
Gaps	10	14	5	957		
Range of Gaps	1–15	1–49	1–16			
Excluded	none	none	none	none	none	none
	56	30	36	40		
Length	1035	1180	714	2929	48	2977
Informative characters	33	46	82	161	45	209
Variable characters	127	236	207	570	48	618
Trees	35458	87213	7259	2301	591	555
Steps	71	77	169	378	278	1177
CI (consistency index)	59	69	72	57	30	62
RI (retention index)	69	90	84	74	57	59
BB (branch and bound) above 75%	2	2	9	7	0	6

### Combined molecular MP analysis

Following the methods outlined by Mason-Gamer and Kellogg (1996) and applied by Eldenäs and Linder (2000), the data sets were considered combinable. Within each gene analysis, *trnL-trnF*, *Rpl32-trnL* and ITS, the genus was monophyletic with 100% bootstrap support. Among the molecular trees there were no conflicting nodes with bootstrap support greater than 75%; therefore congruence exists between the data sets and a combined molecular analysis was completed.

**Figure 2.**
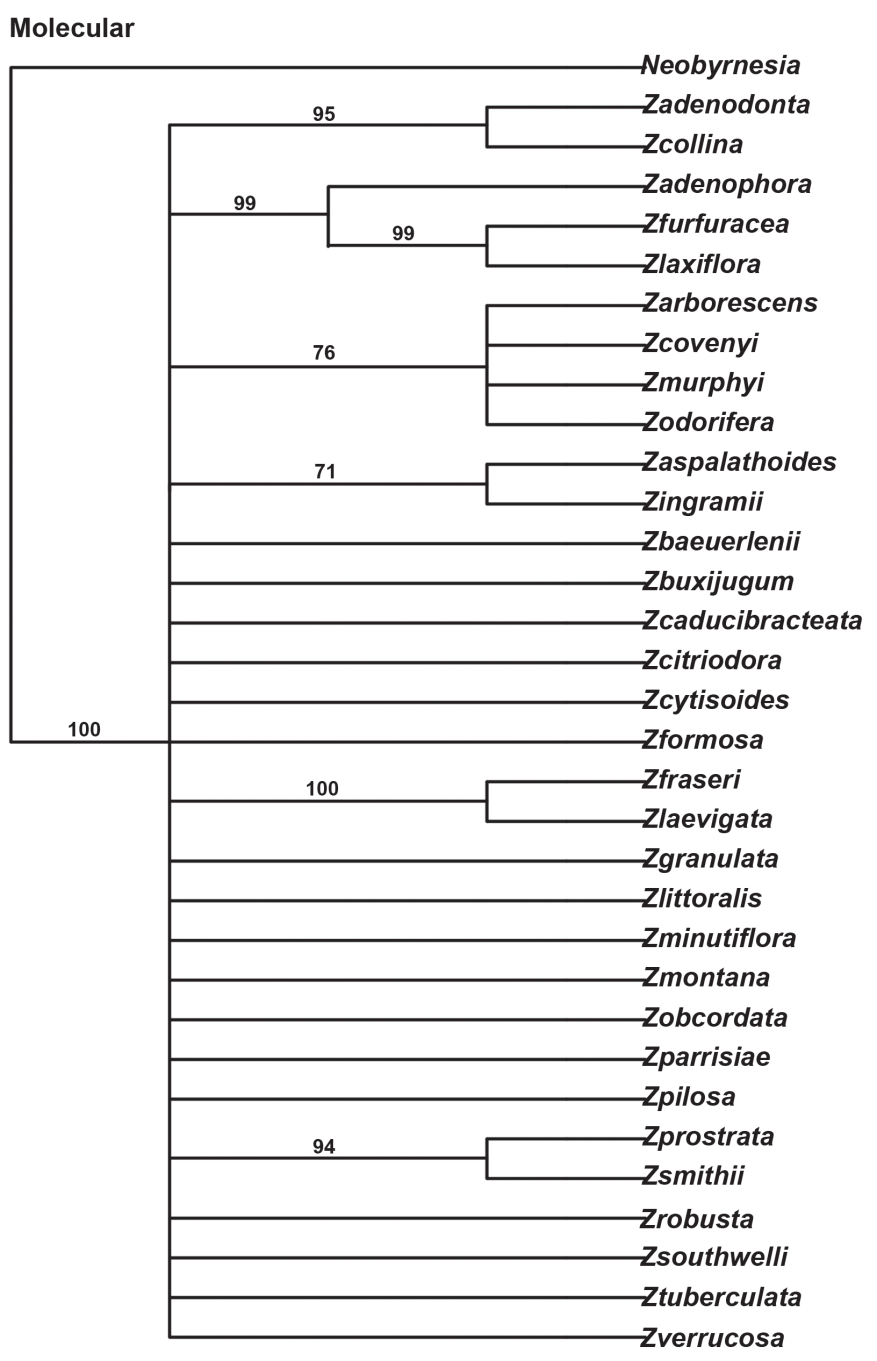
The strict MP consensus tree (L. = 749 steps, CI = 0.57, RI = 0.39) obtained from all molecular data. Numbers above nodes are bootstrap values.

Multiple sequence alignment of *Zieria* and *Neobyrnesia* resulted in a matrix of 2929 characters, of which (32.7%) include at least one accession with a gap. Mean percentage G + C content is 40%. Of the 2929, 570 (19.5%) were variable and 161 (5.5%) were parsimony informative. The analysis recovered 2,301 equally optimal trees of 378 steps (CI = 0.57, RI = 0.74; Fig. 2 majority rule tree).

### *Zieria* was supported as monophyletic in the strict consensus trees (BS 100)

Internally, *Zieria* consists of mainly a polytomy except for several minor clades with bootstrap support greater than 75%. Clades that contain bootstrap support greater than 75% starting from the base of the tree include: 1) a clade containing *Zieria prostrata* and *Zieria smithii* (BS 94%); 2) a clade containing *Zieria fraseri* and *Zieria laevigata* (BS 100%); 3) a clade containing a polytomy of *Zieria arborescens*, *Zieria covenyi*, *Zieria murphyi* Blakely and *Zieria odorifera* A. Cunn. (BS 76%); 4) a clade containing *Zieria furfuracea* and *Zieria laxiflora* (BS 99%) sister to *Zieria adenophora* (BS 99%); and 5) a clade containing *Zieria collina* and *Zieria adenodonta* (BS 95%).

### Morphological-based MP analysis

Of the 48 characters constituting the non-molecular dataset, 48 were variable and 45 (93.8%) were parsimony-informative. The analysis recovered 591 equally optimal trees of 278 steps (CI = 0.30, RI = 0.57). *Zieria* was monophyletic in the strict consensus of these trees (BS 100%). The in-group topology consisted of a large grade with only one clade that contained bootstrap support greater than 75% (*Zieria laxiflora* and *Zieria laevigata* (BS 75%)).

### Combined molecular and morphological data

Following the methods outlined by Mason-Gamer and Kellogg (1996), the molecular and morphological data sets contained only one potential hard conflict between a clade containing *Zieria fraseri* and *Zieria laevigata* (BS 100%) in the molecular data set and a clade containing *Zieria laxiflora* and *Zieria laevigata* (BS 75%) sister to *Zieria fraseri* in the morphology data set. The positions of these three taxa have interchanged among the three separate molecular data sets and this is reflected in the morphology matrix having all three grouped together. The conflict appears to be due to a lack of resolution within the independent molecular dataset or that some of the morphological characters are homoplasious; therefore congruence exists between the data sets and a combined analysis was completed.

**Figure 3.**
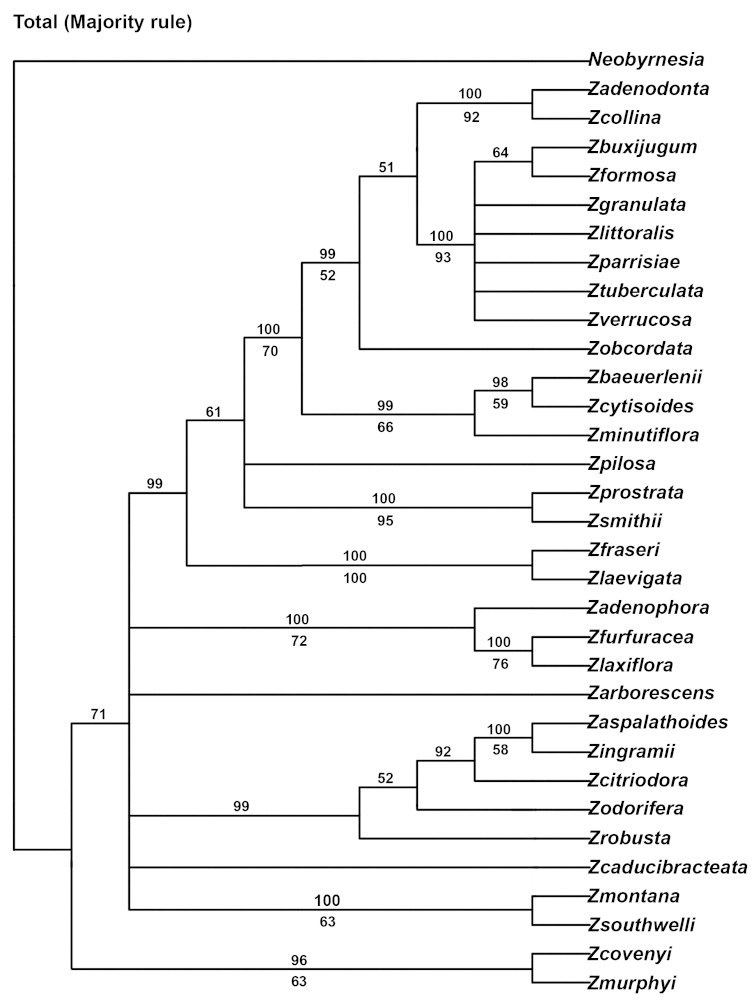
MP majority rule consensus tree using molecular and morphological data. Numbers below nodes are bootstrap values.

Multiple sequence alignment of *Zieria* and *Neobyrnesia* resulted in a matrix of 2977 characters, of which 28% include at least one accession with a gap. Of the 2977 positions constituting the aligned sequences, 618 (%) were variable and 209 (%) were parsimony informative. The analysis recovered 555 equally optimal trees of 1177 steps (CI = 0.62, RI = 0.59; Fig. 3 majority rule tree).

*Zieria* was supported as monophyletic in the strict consensus trees (BS 100).

*Zieria* consists mainly of grades except for several minor clades with bootstrap support greater than 75%. Clades that contain bootstrap support greater than 75% starting from the base of the clade include: 1) a clade containing *Zieria furfuracea* and *Zieria laxiflora* (BS 76%); 2) a clade containing *Zieria fraseri* and *Zieria laevigata* (BS 100%); 3) a clade containing *Zieria prostrata* and *Zieria smithii* (BS 95%); 4) a clade containing a polytomy of (*Zieria buxijugum* J.D. Briggs & J.A. Armstr., *Zieria formosa* J.D. Briggs & J.A. Armstr.), *Zieria granulata* C. Moore ex Benth., *Zieria littoralis* J.A. Armstr., *Zieria parrisiae* J.D. Briggs & J.A. Armstr., *Zieria tuberculata* J.A. Armstr., and *Zieria verrucosa* (BS 93%); and 5) a clade containing *Zieria collina* and *Zieria adenodonta* (BS 92%).

### Bayesian analysis of molecular and morphological data

**Figure 4.**
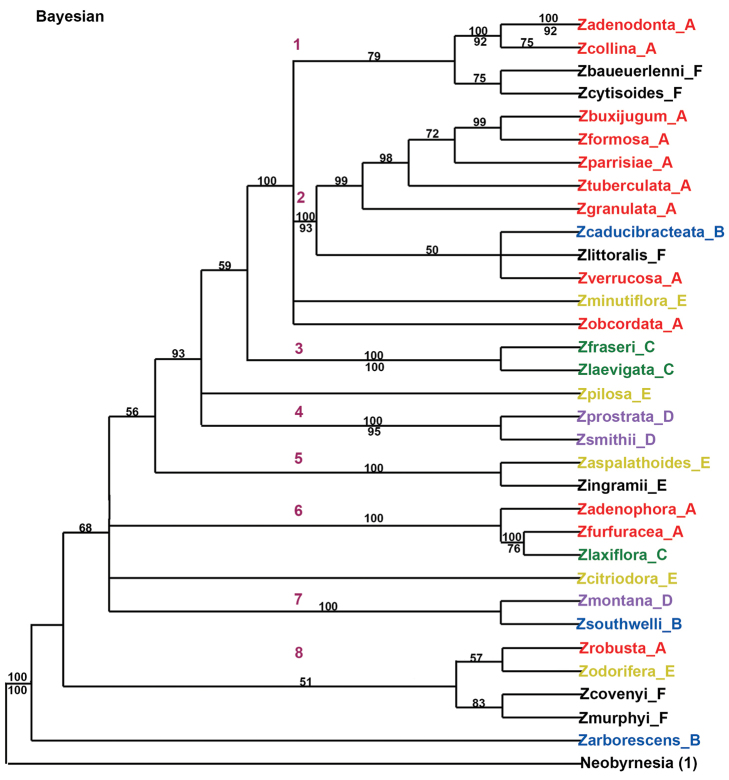
Bayesian majority rule consensus tree using molecular and morphological data. Numbers above the nodes are posterior probability values. **A–F** at the end of the taxa names corresponds to Armstrong (2002) classification system. The 1-8 listed on the tree corresponds to this study's finding.

In the Bayesian analysis (Fig. 4) *Zieria* is resolved as a monophyletic group, which consists mainly of a grade with the following clades containing posterior probability values higher than or equal to 95%: 1) a clade containing *Zieria montana* and *Zieria southwelli* (100); 2) a clade containing ((*Zieria furfuracea* and *Zieria laxiflora* (100)) (*Zieria adenophora* (100))); 3) a clade containing *Zieria aspalathoides* and *Zieria ingramii* (100); 4) a clade containing *Zieria prostrata* and *Zieria smithii* (100); 5) a clade containing *Zieria fraseri* and *Zieria laevigata* (100); 6) a clade containing a grade of ((*Zieria buxijugum*, *Zieria formosa* (99)), *Zieria parrisiae*, *Zieria tuberculata* (98), *Zieria granulata* (99)), sister to ((*Zieria littoralis*, *Zieria caducibracteata* J.A. Armstr., and *Zieria verrucosa*)) (100); 7) a clade containing *Zieria collina* and *Zieria adenodonta* (100); and 8) clades in number 6 and 7 along with *Zieria minutiflora* and *Zieria obcordata* (100). There are no hard conflicts between the supported clades of the Bayesian and the parsimony topologies; in fact they are very similar except for the position of *Zieria caducibracteata*, which is just a matter of resolution. An examination of the 48 morphological characters revealed no unambiguous synapomorphies.

### Conservation

This study examined 15 of the 21 endangered or vulnerable species found in *Zieria* for similarity in their distribution patterns and for the number of bp changes within all three genes inside clades that had strong posterior probabilities.

The first clade containing *Zieria adenodonta* and *Zieria collina* (BPP 100 and BS 92%) have similar distribution patterns, however two of the three genes indicated had numerous bp changes (over 10 bps), indicating the taxa are distinct species.

The second clade contains eight species, one species being *Zieria buxijugum* (BPP 100 and BS 93%), and although the species all occurred mostly in the southeastern territory (New South Wales, Victoria and Tasmania), they had numerous bp changes between taxa.

Within the clade consisting of *Zieria aspalathoides*, and *Zieria ingramii* (BPP 100), there is distributional overlap, however there are over 30 bp changes among the taxa.

Although *Zieria adenophora* has a non-overlapping distribution pattern from *Zieria furfuracea*, and *Zieria laxiflora*, the latter two taxa are very similar in distribution pattern. All three taxa have numerous bp differences, however *Zieria furfuracea* and *Zieria laxiflora* only had 2 solid bp differences.

The third clade consisted of *Zieria prostrata* and *Zieria smithii* (BPP 100 and BS 76%) these taxa have non-overlapping distribution patterns and two of the three genes had numerous bp changes (over 10 bps).

*Zieria covenyi* and *Zieria murphyi* (BPP 83), are from the same area and only had 3 bp changes among all three genes.

## Discussion

### Monophyly of *Zieria* and its closest relatives

We assembled a *trnL-F* dataset including 44 taxa of Rutaceae to determine the outgroup relationship of *Zieria* (Fig. 1). Based on this analysis six species of *Zieria* form a strongly supported clade with *Neobrynesia* (BS 94%). The monophyly of *Zieria* is also suggest by Bayly et al. (2013) and Appelhans et al. (2014). Bayly et al. (2013) using only *rbcL* and *atpB* also included *Zieria chevalieri* from New Caledonian, the only disjunct species within *Zieria* to support not only the monophyly of *Zieria* but also the outgroup relationship with *Neobrynesia*.

Sister to this grouping is a clade containing the following taxa: *Medicosma*, *Euodia*, *Boronia*, *Sarcomelicope* and *Melicope* (see results for BS values and clade arrangements). We therefore used *Neobrynesia* as the outgroup for this study. Armstrong (2002), using morphological features, found that *Zieria*, together with *Boronia* s. l., *Brombya*, *Medicosma*, *Neobyrnesia*, and *Euodia* s. s., formed a distinct clade that is characterized by the presence of foliar sclereids. Although we did not include a species of *Brombya* the remaining members of the above group, plus *Sarcomelicope* and *Melicope*, are represented in the clade.

### Circumscription of *Zieria*

Both independent and combined analyses of the molecular and morphological data supported the monophyly of *Zieria* (Figs 2, 3 and 4), as previously postulated by Armstrong (2002). The present study examined forty-eight morphological characters, including vegetative, floral, and fruit features (Armstrong 2002). Only one character, leaves palmately trifoliolate, provided a synapomorphy for *Zieria* (excluding *Zieria murphyi*). Other morphological characters that had been used to define the genus were examined (e.g. opposite leaves, 4-merous flowers, free petals, four stamens, free filaments, four-lobed disc and dehiscent fruits). Many of these morphological characters (e.g. opposite leaves, 4-merous flowers, four stamens, free filaments, and dehiscent fruits) that were used to define the genus are also found in the outgroup *Neobyrnesia* and in other genera of Australasian Rutaceae, and therefore, are not generic synapomorphies of *Zieria* (Armstrong and Powell 1980). The only other potential synapomorphy of *Zieria* is the intrafloral disc with “distinct antesepalous lobes”, which in *Neobyrnesia* is entire. This study confirms the need to identify additional morphological characters that provide synapomorphies for classification at the generic level.

### Circumscriptions of the six major groups of *Zieria*

On the basis of Armstrong’s (2002) non-molecular phylogenetic study, six major taxon groups were defined for *Zieria*. The MP and the Bayesian analyses of the combined non-molecular and molecular datasets indicate a lack of support for any of these six groups (see Table 1 and Figs 2, 3 and 4).

The MP trees (strict-consensus trees from the independent, the combined molecular, and the non-molecular datasets) are poorly resolved and thus do not allow conclusive evaluation of the classification of Armstrong’s (2002) six taxon groups. The Bayesian tree from the combined molecular and morphological datasets provides groupings with high support; therefore this dataset is used to discuss these relationships (Fig. 4).

Characters that support the six major taxon groups defined by Armstrong (2002) are as follows:

Group A contains 14 species and is characterized by having distinctly tuberculate younger branches, peduncles, petioles, midveins, and fruits.

Group B contains five species. The characteristics include younger branches slightly ridged or terete, primary inflorescence bracts boat-shaped and deciduous leaving a scar, and the abaxial surface of the calyx lobes with stellate hairs.

Group C consists of four species defined by having younger branches distinctly ridged with prominent glabrous leaf decurrencies, lower lamina surface velvet like, midveins glabrous with pellucid glands, inflorescences equal to or longer than the leaves, apex of calyx lobes curved inward adaxially, anthers prominently sharply pointed, and fruits with pellucid glands.

Group D comprises four species with the following characteristics: younger branches distinctly ridged with prominent glabrous leaf decurrencies; lower lamina surface glabrous and with pellucid glands that turn black on drying and become sunken; petiole either with pellucid glands or tuberculate; midvein glabrous with pellucid glands; and fruit with pellucid glands.

Group E is composed of eight species with younger branches densely pubescent, upper lamina surface with simple hairs, lamina lower surface and midvein hirsute, filaments warty towards the apex, anthers prominently sharply pointed, ovary pubescent, cocci sharply pointed, and fruits glabrous or pubescent.

Group F, the final group, consists of seven species. The characteristics include upper lamina surfaces that are velvet like, inflorescences equal to or longer than the leaves, primary bracts that are boat-shaped and fruits that are pubescent.

In examining the Bayesian clade the following three mixed clades indicate that none of Armstrong’s (2002) groups are monophyletic (Fig. 4). 1) *Zieria montana* from Group D forms a sister grouping with *Zieria southwelli* from Group B (BPP 100). 2). *Zieria furfuracea* from Group A forms a sister grouping with *Zieria laxiflora* from Group C (BPP 100). 3). *Zieria minutiflora* (F. Muell.) Domin from Group E forms a well-supported polytomy with taxa from Groups A, B, and F (BPP 100).

### Tentative new groups for *Zieria*

On the basis of the combined Bayesian analysis based on three genes (two-cholorplast and one-nuclear) and a morphological matrix (48 features), eight major taxon groups are distinguishable within *Zieria*. All of these informal groups, except for Groups 1 and 8, correspond to the clades with posterior probability values of 100 (Fig. 4). The make-up of these Groups are as follows:

The examination of the 48 morphological characters within the Bayesian tree revealed no unambiguous synapomorphies. However, sets of morphological synapomorphies in combination provide unique groups of characters to define a clade.

*Zieria cytisoidesGroup 1*: four species — *Zieria adenodonta*, *Zieria baeuerlenii* J.A. Armstr., *Zieria collina*, and *Zieria cytisoides* Sm. This group contained the following synapomorphies: young branches densely pubescent and abaxial lamina surface not tuberculate.

*Zieria granulataGroup 2*: eight species — *Zieria buxijugum*, *Zieria caducibracteata*, *Zieria formosa*, *Zieria granulata*, *Zieria littoralis*, *Zieria parrisiae*, *Zieria tuberculata*, and *Zieria verrucosa*. Morphological characters that were found to be synapomorphic for this clade include: abaxial lamina surface and midvein tuberculate.

*Zieria laevigataGroup 3*: two species — *Zieria fraseri* and *Zieria laevigata*. These taxa had a number of morphological synapomorphies including: young branches, petioles and midveins not tuberculate, lamina suface and filaments pubescent, and calyx lobes glaucous and apex inflexed adaxially.

*Zieria smithiiGroup 4*: two species — *Zieria prostrata* and *Zieria smithii*. Morpholoigcal characters that were found to be synapomorphic for this clade include: lamina surface and peduncles glabrous, lamina surface without black pellucid glands, midveins with pellucid oil glands, and inflorescences containing 10–50 flowers.

*Zieria aspalathoidesGroup 5*: two species — *Zieria aspalathoides* and *Zieria ingramii*. Several morphogical characters are shared by these taxa such as: young branches distinctly ridged and densely pubescent, lamina surface with simple hairs, lamina margins revolute, filaments prominently dilated at base and anthers slightly apiculate.

*Zieria furfuraceaGroup 6*: three species — *Zieria adenophora*, *Zieria furfuracea*, and *Zieria laxiflora*. Only one morphological synapomorphy was found for this grouping: filaments not prominently dilated at base.

*Zieria montanaGroup 7*: two species — *Zieria montana* and *Zieria southwelli*. One synapomorphy was found for these taxa: pubescence consisting of stellate trichomes.

*Zieria robustaGroup 8*: four species — *Zieria covenyi*, *Zieria murphyi*, *Zieria odorifera* and *Zieria robusta* Maiden & Betche. This group had several synapomorphies including the lamina surface containing pellucid oil glands, and the few flowered inflorescences being equal to or longer than the leaves.

Because of the lack of resolution, five taxa, *Zieria citriodora*, *Zieria arborescens*, *Zieria minutiflora*, *Zieria obcordata* and *Zieria pilosa*, will remain unplaced until additional studies are completed. DNA for the following species were not examined and therefore these taxa will not be placed into groups until sequencing and analysis is completed: *Zieria chevalieri*, *Zieria floydii*, *Zieria hindii*, *Zieria involucrata*, *Zieria lasiocaulis*, *Zieria obovata*, *Zieria oreocena*, *Zieria rimulosa*, *Zieria robertsiorum*, and *Zieria veronicea*. Although six of the eight groups have strong posterior probabilities the relationships between these clades remain uncertain. The monophyly of the genus and of six of these groups appears unamibiguous; however, additional molecular and morphological studies are needed to further define the groupings and internal relationships.

### Endangered taxa and conservation issues

Many *Zieria* taxa are considered endangered or vulnerable (Briggs and Leigh 1988, 1996; EPBC Act; current website http://www.environment.gov.au/cgi-bin/sprat/public/spratlookupspecies.pl?name=zieria&searchtype=Wildcard). Of the 51 taxa recognized by Armstrong (2002), the following 21 are considered endangered or vulnerable: *Zieria adenophora*, *Zieria baeuerlenii*, *Zieria bifida*, *Zieria buxijugum*, *Zieria citriodora*, *Zieria collina*, *Zieria convenyi*, *Zieria floydii*, *Zieria formosa*, *Zieria granulata*, *Zieria ingramii*, *Zieria involucrae*, *Zieria lasiocaulis*, *Zieria murphyi*, *Zieria obcordata* A. Cunn., *Zieria obovata*, *Zieria parrisiae*, *Zieria prostrata*, *Zieria rimulosa*, *Zieria verrucosa*, and *Zieria tuberculata*.

This study examined 15 of the 21 endangered or vulnerable taxa found in *Zieria* for similarity in their distribution patterns and for the number of bp changes within all three genes inside clades that had strong posterior probabilities.

*Zieria covenyi* and *Zieria murphyi* (BPP 83), are from the same area and only had 3 bp changes among all three genes. Both taxa have several solid morphological differences such as leaves pubescent or glabrous, inflorescence numerous or few and filaments dilated or not dilated respectively. Because of these solid morphological differences these species appear distinct.

*Zieria furfuracea*, and *Zieria laxiflora*, (BPP 100) were very similar in pattern and had only 2 bp differences. Once again an examination of the non-molecular features revealed a number of differences such as the leaves having stellate-pubescence vs. being glabrous; flowers ranging from 21–125 vs. commonly 9; petals valvate vs. imbricate; and flowering from spring to early summer vs. late winter to spring, to name a few.

Taxa in clades with strong posterior probabilities, with similar distribution patterns and low genetic variation, need to be closely examined before conservation management decisions are made to assure that they are unique species.

## Conclusion

*Zieria* as currently circumscribed (Armstrong 2002) is monophyletic. This is supported by the molecular phylogenetic analysis and by one morphological synapomorphy: distinct antesepalous lobes of the gynoecium. This study found that the previous six species groups considered by Armstrong (2002) are not monophyletic, and confirmed that *Neobyrnesia* is the closest relative to *Zieria*. The analyses identified eight groups within *Zieria* and six of the eight groups have strong posterior probabilities.

Based on the number of informative characters and the number of branches with supported, ITS is an excellent candidate for higher-level analysis. In addition, ITS produced very few alignment difficulties within the ingroup and outgroup, and its tree topology remained consistent with that of the other genes.

Of the 32 taxa used in this study, 21 are considered endangered or vulnerable according to the EPBC. Several taxa grouped together and formed clades with strong posterior probabilities. Further examination revealed that two of these groups had similar distribution patterns and low genetic variation but solid differences in non-molecular characters. The taxonomic relationships of these taxa should be closely examined as further conservation management decisions are made.

The phylogenetic analysis presented here provides the first study within *Zieria* using both chloroplast and nuclear datasets, as well as a morphological dataset. Topics to be addressed in a future study include the determination of tribal and subtribal groupings and the use of additional taxa and genes to elucidate the biogeographic history of the genus.

## Appendix 1

**Appendix 1:** *Zieria* species sequenced for the present study, with *Neobyrnesia* as outgroup. Collection data for accession vouchers and GenBank accession numbers are given below (see Materials and Methods). The country of origin for all specimens is Australia and specimens were collected from the associated botanical garden^1^.

Species	Herbarium voucher	GenBank Accession Numbers		
		*rpl32-trnL*	*trnL-trnF*	*ITS*
*Neobyrnesia suberosa* J.A. Armstr.	*R. Mueller s.n. Dec. 2 1982* (CBG-8316286)	EU281888	EU281921	EU281855
*Zieria adenodonta* (F. Muell.) J.A. Armstr.	*F. A. Zich 453* (CANB 653334)	EU281889	EU281922	EU281856
*Zieria adenophora* Blankly	*J. A. Armstrong et al. 5097a* (CBG-8805884)	EU281890	EU281923	EU281857
*Zieria arborescens* Sims.	*I. R. Telford 3134* ^*^(CBG-54528) *S. R. Donaldson & S. Golson 3594* ^**^(CANB-748530)	EU281891	EU281924	EU281858
*Zieria aspalathoides* A. Cunn. ex Benth	*D. L. Jones & C.H. Broers 7814* (CBG-9109508	EU281892	EU281925	EU281859
*Zieria baeuerlenii* J.A. Armstr.	*S. Donaldson 111A* CBG-9104885	EU281893	EU281926	EU281860
*Zieria buxijugum J.D. Briggs* & *J.A. Armstr.*	*M. Parris* et al. *9018a* CBG-8602343	EU281894	EU281927	EU281861
*Zieria caducibracteata* J.A. Armstr.	*J. A. Armstrong & R. Coveny 744* CBG-8208729	EU281895	EU281928	EU281862
*Zieria citriodora* J.A. Armstr.	*I.R. Telford & S. Corbett 7346* CBG- 8001161	EU281896	EU281929	EU281863
*Zieria collina* C.T. White	*M. Parris 8847* (CBG- 8413675	EU281897	EU281930	EU281864
*Zieria covenyi* J.A. Armstr.	*P. Beesley et al. 285* (CBG-8411672)	EU281898	EU281931	EU281865
*Zieria cytisoides* Sm.	*F. A. Zich 405* CANB-643984 (CANB-629784)	EU281899	EU281932	EU281866
*Zieria formosa* J.D. Briggs & J.A. Armstr.	*M. Parris & N. Fisher 9151a* CBG-8604998	EU281900	EU281933	EU281867
*Zieria fraseri* Hook.	*I.R. Telford & S. Donaldson 12120* (CANB-9613250)	EU281901	EU281934	EU281868
*Zieria furfuracea* R.Br. ex Benth.	*A. M. Lyne et al. 2143* (CBG-9705354)	EU281902	EU281935	EU281869
*Zieria granulata* C. Moore ex Benth.	*K. Mills 2A* CBG-8501509 (CBG-9505133)	EU281903	EU281936	EU281870
*Zieria ingramii* J.A. Armstr.	*K. M. Groeneveld 89* A CBG-8800001	EU281904	EU281937	EU281871
*Zieria laevigata* Bonpl.	*F. A. Zich 448* CANB 653329	EU281905	EU281938	EU281872
*Zieria laxiflora* Domin	*S. Fethers et al. 11* (CANB-617460)	EU281906	EU281939	EU281873
*Zieria littoralis* J.A. Armstr.	*M. Parris & N. Fisher 9240* CBG-8703977	EU281907	EU281940	EU281874
*Zieria minutiflora* (F. Muell.) Domin	*P. Beesley & P. Ollerenshaw 959* CBG-8604299	EU281908	EU281941	EU281875
*Zieria montana* J.A. Armstr	*F. A. Zich 462* CANB 653343	EU281909	EU281942	EU281876
*Zieria murphyi* Blakely	*A. M. Lyne et al. 325* CBG-9101073	EU281910	EU281943	EU281877
*Zieria obcordata* A. Cunn.	*J. D. Briggs 2376* CANB-389372	EU281911	EU281944	EU281878
Zieria odorifera J.A. Armstr. subsp. williamsii Duretto & P.I. Forst.	*I. Southwell H85-039* CBG-8505944	EU281912	EU281945	EU281879
*Zieria parrisiae* J.D. Briggs & J.A. Armstr.	*M. Parris 9145B* CBG-8604990	EU281913	EU281946	EU281880
*Zieria pilosa* Rudge	*D. L. Jones & C. Broers 6063* CBG-9010362)	EU281914	EU281947	EU281881
*Zieria prostrata* J.A. Armstr.	*S. Myers ANGB 2134a* (CBG-8802463)	EU281915	EU281948	EU281882
*Zieria robusta* Maiden & Betche	*M. D. Crisp 4397* CBG-7809037	EU281916	EU281949	EU281883
*Zieria smithii* Andrews	*S. Pedersen 16* CBG-9705152	EU281917	EU281950	EU281884
*Zieria southwelli* J.A. Armstr.	*I. R. Telford 3298* CBG- 54531	EU281918	EU281951	EU281885
*Zieria tuberculata* J.A. Armstr.	J. D. Briggs 2344 CANB 387032	EU281919	EU281952	EU281886
*Zieria verrucosa* J.A. Armstr.	*P. Beesley & P. Ollerenshaw 970A* CBG-8604310	EU281920	EU281953	EU281887

1 The herbarium holdings of the Australian National Botanic Gardens (CBG) were combined in 1993 with those of the Australian National Herbarium (CANB) as part of the Centre for Plant Biodiversity Research, now the Centre for Australian National Biodiversity Research, (CANB), was adopted as the herbarium abbreviation for the combined collections; however, specimens originally from CBG continue to be cited as CBG.

* Asterisks indicate which sample was used for each gene.
